# Editorial: New Insights on Basic and Clinical Aspects of EEG and MEG Connectome

**DOI:** 10.3389/fnhum.2018.00232

**Published:** 2018-06-05

**Authors:** Ryouhei Ishii, Roberto D. Pascual-Marqui, Leonides Canuet, Jing Xiang, William C. Gaetz

**Affiliations:** ^1^Department of Psychiatry, Osaka University Graduate School of Medicine, Suita, Japan; ^2^Neuroscience Center, Ashiya Municipal Hospital, Ashiya, Japan; ^3^The KEY Institute for Brain-Mind Research, University of Zurich, Zurich, Switzerland; ^4^Neuropsychiatry, Kansai Medical University, Moriguchi, Japan; ^5^Department of Cognitive, Social and Organizational Psychology, La Laguna University, Tenerife, Spain; ^6^Cincinnati Children's Hospital Medical Center, Cincinnati, OH, United States; ^7^Children's Hospital of Philadelphia, Philadelphia, PA, United States

**Keywords:** EEG, MEG, connectome, LORETA, default mode network (DMN)

Previous studies have shown that, in electroencephalography (EEG) and magnetoencephalography (MEG), cortical oscillations in specific frequency bands (i.e., delta, theta, alpha, beta, and gamma) are functionally related to cognitive processing and behavior, and that abnormal patterns correlate with pathophysiological processes of neuropsychiatric disorders. Although these results have provided useful insights for neural communication in the human brain, they appeared to be highly speculative based on the level of EEG and MEG analyses performed (simple averaging method, wavelet analysis on the sensor level, etc.). The development of new analysis methods for EEG and MEG signals has allowed researchers to depict the information code of brain networks, by precisely localizing sources of oscillatory activity related to brain functions or pathological processes. However, this kind of approach, characterizing brain activity purely in terms of anatomically segregated responses, is not sufficient to explain the pathophysiology of complex neuropsychiatric disorders or the mechanisms underlying cognitive functions.

Recent advances in the neuroimaging field areas allow us to visualize the aggregate of neural connections at the macroscopic level within the brain, the so-called “connectome.” In order to promote the development of the neurophysiological investigation of connectome of brain oscillations, this e-book aims at bringing together contributions from researchers in basic and clinical neuroscience using EEG and MEG connectome analysis. The most important focal point will be to address the functional roles of connectome of brain oscillations in contributing to understandings of higher cognitive processes in normal subjects and pathophysiology of psychiatric diseases.

The included papers can be roughly divided into three groups as follows: the first group of papers both tried to expand the boundaries of brain connectome analysis by applying new statistic solvers on exact low resolution electromagnetic tomography (eLORETA) analysis of EEG. Pascual-Marqui et al. who founded LORETA himself, presented namely the “isolated effective coherence” (iCoh) obtained from eLORETA, which consists of estimating the partial coherence under a multivariate autoregressive model, followed by setting all irrelevant associations to zero, other than the particular directional association of interest. They elucidated the direct directed connection of alpha activity from the posterior cingulate to all other regions during eyes closed and theta-alpha activity from the anterior cingulate to other frontal regions during eyes open in human brain. Aoki et al. applied eLORETA-ICA analysis to resting-state EEG data in 80 healthy subjects using five frequency bands (delta, theta, alpha, beta and gamma band) and found five resting-state-independent-networks in alpha, beta and gamma frequency bands. Thatcher et al. analyzed the phase shift and lock duration of 3-dimensional current sources in 14 Brodmann areas located in the DMN using LORETA in the delta frequency band by using the Hilbert transform between all pairs of Brodmann areas. By depicting an inverse relation of phase shift and lock durations in an exponential way with distance between Brodmann areas, they showed a tremendous view of anatomical hubs in the brain behave like a “shutter” that opens and closes at specific durations as nodes of brain network. These cutting-edge methodologies were often applied in the recent publications and cited almost 60 times in these four years in total.

The application of these connectivity analysis for basic cognitive studies was described in the following papers—the focus of the second group. To examine the relationship between functional connectivity and performance of brain-machine interfaces (BMIs), Sugata et al. analyzed the correlation coefficient between performance of neural decoding and functional connectivity over the whole brain using. They suggest that use of the strength of functional connectivity between M1 and motor association areas has the potential to improve the performance of BMIs to perform real and imagined movements. Asakawa et al. evaluated the effect of different anxiety states on information processing as measured by EEG using emotional stimuli on a smartphone. They found that the propagation speed of the low anxiety group at the medial coronal for resting stimuli for all time segments was higher than those of high anxiety group and suggested that neural information processes concerning emotional stimuli differ based on current anxiety state. Ishii et al. used spatially filtered MEG and permutation analysis to precisely localize cortical generators of the magnetic counterpart of frontal midline theta rhythm (Fmθ) and found the dorsal anterior cingulate and adjacent medial prefrontal cortex as the generetors of Fmθ and gamma event-related synchronization in right parietal regions subserving basic numerical processing and number-based spatial attention. They suggested that these multiple oscillatory activities might interact each other to proceed these cognitive tasks in the brain. Leiken et al. reported high-frequency brain signals in the developing brain using time-frequency analysis along with beamforming methods on MEG data. They suggested that the developing brain generates high-frequency signals that can be detected with the non-invasive technique of MEG. These studies showed several type of connectivity among certain brain areas in multiple frequency bands such as theta, alpha, beta, gamma bands related to various types of cognitive process and behavior.

The third group contains the new research proposal from Ibanez et al. assessing the combination of basal resting state influences together with the ongoing activity during a task and its evoked neural response to characterize brain dynamics changes related to preclinical Alzheimer's disease and the review article from Miki and Kakigi introducing their own three studies that focused on facial movements.

In summary, this ebook presented novel methodologies and various applications of neurophysiological connectome analysis. As a result, these papers were cited more than 120 times in these 4 years in total and threw light and impact on new directions for investigating the connectome of human brain.

## Author contributions

RI and LC discussed about this research topic and wrote the manuscript. RP-M, JX, and WG gave significant advise and helped the editing the manuscript.

### Conflict of interest statement

The authors declare that the research was conducted in the absence of any commercial or financial relationships that could be construed as a potential conflict of interest.

